# Supplementary material for: Ancient mechanisms for the evolution of the bicoid homeodomain's function in fly development
Source: eLife. 2018 Oct 9;7:e34594. doi: 10.7554/eLife.34594 (PMC6177261; doi:10.7554/eLife.34594)
Supplement: Supplementary file 1. — For each sequence in the alignment, the genus and species name, amino acid sequence, and abbreviation used in the phylogeny are shown. [file elife-34594-supp1.docx]

**Supplemental File 1. Extant homeodomain sequences used for phylogenetic analysis and ancestral reconstruction.**

| **Species** | **Abbrevi-ation** | **Sequence** |
| --- | --- | --- |
| *Rhajio tringarius* | RtrZen | LKRSRTAFTSVQLIELENEFKKQRYLFRPRRIELALQLKLSERQIKVWFQNRRMKEKKDQ |
| *Lonchoptera lutea* | LluZen | LKRSRTAFTSLQLLELEREFHINKYLCRPRRIEIAQRLSLSERQVKIWFQNRRMKSKKDS |
| *Episyrphus balteatus* | EbaZen | CKRARTAFSSNQLIQLEREFHTNKYLCRPRRIEISQRLELSERQVKIWFQNRRMKSKKDA |
| *Platypeza consobrina* | PcoZen | SKRSRTAFTSLQLIELEREFHINKYLCRPRRIEISQRLVLSERQVKIWFQNRRMKNKKHQ |
| *Aedes aegypti* | AaeZen | TKRSRTAFTSQQLVELEKEFRLNRYLCRPRRIEIATKLALTERQIKIWFQNRRMKHKKDI |
| *Culex quinquefasciatus* | CquZen | TKRSRTAYTGIQLQALEKEFSHNRYLCRPRRIEIATKLGLSERQIKIWFQNRRMKHKKES |
| *Drosophila melanogaster* | DmeZen | LKRSRTAFTSVQLVELENEFKSNMYLYRTRRIEIAQRLSLCERQVKIWFQNRRMKFKKDI |
| *Anopheles gambiae* | AgaZen | TKRSRTAFTSSQLVELEKEFHSNRYLCRPRRIELTRKLALTERQIKIWFQNRRMKHKKES |
| *Bombyx mori* | BmoZen | TKRARTAYTSSQLVELENEFHQNRYLCRPRRIELANYLQLSERQIKIWFQNRRMKYKKDN |
| *Danaus plexippus* | DplZen | TKRARTAYTSSQLVELENEFHQNRYLCRPRRIELANYLQLSERQIKIWFQNRRMKYKKDN |
| *Oncopeltus Fasciatus* | OfaZen | AKRARTAYTSIQLVELEKEFHFNRYLCRPRRIEMATQLRLSERQIKIWFQNRRMKYKKER |
| *Thermobia domestica* | TdoHox3 | AKRARTAYTSAQLVELEKEFHFNRYLCRPRRIEMAALLNLTERQIKIWFQNRRMKYKKEQ |
| *Bombus impatiens* | BimZen | TKRTRTAYTSAQLVELEKEFNRTRYLCRPRRIELAAALSLTERQIKIWFQNRRMKYKKDQ |
| *Apis mellifera* | AmeZen | TKRSRTAYTSVQLVELEKEFTVTKYLCRPRRIELAIALSLTERQIKIWFQNRRMKYKKEQ |
| *Folsomia candida* | FcaHox3 | TKRARTAYTSAQLVELEKEFHYNRYLCRPRRIEMASLLSLTERQIKIWFQNRRMKYKKEL |
| *Daphnia pulex* | DpuHox3 | AKRARTAYTSAQLVELEKEFHFNRYLCRPRRIEMATLLNLTERQIKIWFQNRRMKFKKEQ |
| *Strigamia maritima* | SmaHox3 | NKRSRTAYTQSQLVELEKEFHFNRYLCRPRRVELASMLNLTERQIKIWFQNRRMKNKKIK |
| *Tribolium castaneum* | TcaZen | GKRARTAYTSAQLVELEREFHHGKYLSRPRRIQIAENLNLSERQIKIWFQNRRMKHKKEQ |
| *Tribolium castaneum* | TcaZen2 | GKRARTAYTSSQLVELEREFHRSKYLCRPRRIQMAQNLNLTERQIKIWFQNRRMKFKKEE |
| *Schistocerca gregaria* | SgrZen | SKRARTAYTSQQLIELEKEFSINRYLCRPRRIELAAQLGLTERQIKIWFQNRRMKYKKEK |
| *Haematopota pluvialis* | HplZen | PKRARTAYSSVQLMELEKEFNMGSYLCRPRRIELANKLKLNERQIKIWFQNRRMKHKKSK |
| *Empis livida* | EliZen | IKRPRTAFTSHQLFELENEYKLNEYLSRPRRIEISQRLSLQERNVKVWFQNRRMKQKKDM |
| *Megaselia abdita* | MabZen | TKRSRTAFTSIQLLELENEFKKNKYLNRPRRIEISLRLSLSERQVKIWFQNRRMKSKKDR |
| *Clogmia albipunctata* | CalZen | KKRSRTAYTSYQLVALERAFLKNNYISRPARTFMAKELGLIEKQIKIWFQNRRMKENKSN |
| *Megaselia abdita* | MabBcd | -RRTRTTFTSSQIAELEEYFRQGKYLNNIRLSELTGRLNLGQAQVKIWFKNRRRRFKIEQ |
| *Platypeza consobrina* | PcoBcd | TRRLRTTFTQQQLQELEQEFQINKYVTALRLADITSRLNLANAQVKIWFKNRRRKHKIEE |
| *Lonchoptera lutea* | LluBcd | PRRTRTTFTSAQISKLEQYFNESKYVNASRLAELSGKLNLGNAQVKIWFKNRRRRLRIEQ |
| *Musca domestica* | MdoBcd | PRRTRTTFTSAQIAELEQHFLQGRYLTSSRLAELSAKLTLGTAQVKIWFKNRRRRHKIQS |
| *Calliphora vicina* | CviBcd | PRRTRTTFTSAQIAELEQHFLQGRYLTSSRLAELSAKLALGTAQVKIWFKNRRRRHKIQA |
| *Triceratopyga calliphoroides* | TralBcd | PRRTRTTFTSAQIAELEQHFLQGRYLTSSRLAELSAKLGLGTAQVKIWFKNRRRRHKIQS |
| *Lucilia sericata* | LseBcd | PRRTRTTFTSAQIAELEQHFLQGRYLTSSRLAELSAKLALGTAQVKIWFKNRRRRHKIQS |
| *Drosophila melanogaster* | DmeBcd | PRRTRTTFTSSQIAELEQHFLQGRYLTAPRLADLSAKLALGTAQVKIWFKNRRRRHKIQS |
| *Episyrphus balteatus* | EbaBcd | PRRSRTVFTPDQVAELKYHFMQCNYIRFEKAEQIAAKFSMPVGPVKVWFKNQRRKLKIKE |
