# Supplementary material for: Ancient mechanisms for the evolution of the bicoid homeodomain's function in fly development
Source: eLife. 2018 Oct 9;7:e34594. doi: 10.7554/eLife.34594 (PMC6177261; doi:10.7554/eLife.34594)
Supplement: Supplementary file 2. — At each sequence site, the three amino acid states with the highest mpp are shown for the HDs of AncZB (panel A) and AncBcd (panel B). Red indicates ambiguously reconstructed sites, defined as those that have more than one state with mpp >0.20. [file elife-34594-supp2.docx]

**Supplemental File 2. Reconstructed ancestral sequences and site-specific marginal posterior probabilities (mpp).** At each sequence site, the three amino acid states with the highest mpp are shown for the HDs of AncZB (panel A) and AncBcd (panel B). Red indicates ambiguously reconstructed sites, defined as those that have more than one state with mpp>0.20.

**A)**

| AncZB-HD | | | | | | |
| --- | --- | --- | --- | --- | --- | --- |
| Site | State 1 | mpp 1 | State 2 | mpp 2 | State 3 | mpp 3 |
| 1 | T | 0.404 | L | 0.299 | S | 0.103 |
| 2 | K | 1 |  |  |  |  |
| 3 | R | 1 |  |  |  |  |
| 4 | S | 1 |  |  |  |  |
| 5 | R | 1 |  |  |  |  |
| 6 | T | 1 |  |  |  |  |
| 7 | A | 1 |  |  |  |  |
| 8 | F | 1 |  |  |  |  |
| 9 | T | 1 |  |  |  |  |
| 10 | S | 1 |  |  |  |  |
| 11 | L | 0.712 | I | 0.205 | V | 0.07 |
| 12 | Q | 1 |  |  |  |  |
| 13 | L | 1 |  |  |  |  |
| 14 | I | 0.872 | L | 0.1 | V | 0.027 |
| 15 | E | 1 |  |  |  |  |
| 16 | L | 1 |  |  |  |  |
| 17 | E | 1 |  |  |  |  |
| 18 | R | 0.918 | N | 0.058 | K | 0.023 |
| 19 | E | 1 |  |  |  |  |
| 20 | F | 1 |  |  |  |  |
| 21 | H | 1 |  |  |  |  |
| 22 | I | 0.801 | T | 0.137 | M | 0.034 |
| 23 | N | 1 |  |  |  |  |
| 24 | K | 0.999 | R | 0.001 |  |  |
| 25 | Y | 1 |  |  |  |  |
| 26 | L | 1 |  |  |  |  |
| 27 | C | 1 |  |  |  |  |
| 28 | R | 1 |  |  |  |  |
| 29 | P | 1 |  |  |  |  |
| 30 | R | 1 |  |  |  |  |
| 31 | R | 1 |  |  |  |  |
| 32 | I | 1 |  |  |  |  |
| 33 | E | 1 |  |  |  |  |
| 34 | I | 1 |  |  |  |  |
| 35 | S | 0.927 | A | 0.073 |  |  |
| 36 | Q | 1 |  |  |  |  |
| 37 | R | 0.997 | K | 0.003 |  |  |
| 38 | L | 1 |  |  |  |  |
| 39 | S | 0.921 | A | 0.059 | T | 0.019 |
| 40 | L | 1 |  |  |  |  |
| 41 | S | 0.999 | T | 0.001 |  |  |
| 42 | E | 1 |  |  |  |  |
| 43 | R | 1 |  |  |  |  |
| 44 | Q | 1 |  |  |  |  |
| 45 | V | 1 |  |  |  |  |
| 46 | K | 1 |  |  |  |  |
| 47 | I | 1 |  |  |  |  |
| 48 | W | 1 |  |  |  |  |
| 49 | F | 1 |  |  |  |  |
| 50 | Q | 1 |  |  |  |  |
| 51 | N | 1 |  |  |  |  |
| 52 | R | 1 |  |  |  |  |
| 53 | R | 1 |  |  |  |  |
| 54 | M | 1 |  |  |  |  |
| 55 | K | 1 |  |  |  |  |
| 56 | S | 0.964 | N | 0.033 | H | 0.002 |
| 57 | K | 1 |  |  |  |  |
| 58 | K | 1 |  |  |  |  |
| 59 | D | 1 |  |  |  |  |
| 60 | S | 0.712 | A | 0.119 | Q | 0.088 |
|  |  | 0.970 |  |  |  |  |

**B)**

| AncBcdHD | | | | | | |
| --- | --- | --- | --- | --- | --- | --- |
| Site | State 1 | mpp 1 | State 2 | mpp 2 | State 3 | mpp 3 |
| 1 | P | 1 |  |  |  |  |
| 2 | R | 1 |  |  |  |  |
| 3 | R | 1 |  |  |  |  |
| 4 | T | 1 |  |  |  |  |
| 5 | R | 1 |  |  |  |  |
| 6 | T | 1 |  |  |  |  |
| 7 | T | 1 |  |  |  |  |
| 8 | F | 1 |  |  |  |  |
| 9 | T | 1 |  |  |  |  |
| 10 | S | 1 |  |  |  |  |
| 11 | A | 0.813 | S | 0.184 | T | 0.003 |
| 12 | Q | 1 |  |  |  |  |
| 13 | I | 1 |  |  |  |  |
| 14 | A | 0.999 |  |  |  |  |
| 15 | E | 1 |  |  |  |  |
| 16 | L | 1 |  |  |  |  |
| 17 | E | 1 |  |  |  |  |
| 18 | Q | 0.999 |  |  |  |  |
| 19 | H | 1 |  |  |  |  |
| 20 | F | 1 |  |  |  |  |
| 21 | L | 0.997 | I | 0.001 | M | 0.001 |
| 22 | Q | 0.997 | E | 0.002 |  |  |
| 23 | G | 1 |  |  |  |  |
| 24 | R | 0.997 | K | 0.003 |  |  |
| 25 | Y | 1 |  |  |  |  |
| 26 | L | 1 |  |  |  |  |
| 27 | T | 0.997 | N | 0.001 | S | 0.001 |
| 28 | A | 0.772 | S | 0.227 |  |  |
| 29 | S | 0.678 | P | 0.315 | A | 0.003 |
| 30 | R | 1 |  |  |  |  |
| 31 | L | 1 |  |  |  |  |
| 32 | A | 1 |  |  |  |  |
| 33 | E | 0.959 | D | 0.041 |  |  |
| 34 | L | 1 |  |  |  |  |
| 35 | S | 1 |  |  |  |  |
| 36 | A | 0.997 | G | 0.002 | S | 0.001 |
| 37 | K | 0.997 | R | 0.003 |  |  |
| 38 | L | 1 |  |  |  |  |
| 39 | A | 0.996 | S | 0.002 |  |  |
| 40 | L | 1 |  |  |  |  |
| 41 | G | 1 |  |  |  |  |
| 42 | T | 0.999 | N | 0.001 |  |  |
| 43 | A | 1 |  |  |  |  |
| 44 | Q | 1 |  |  |  |  |
| 45 | V | 1 |  |  |  |  |
| 46 | K | 1 |  |  |  |  |
| 47 | I | 1 |  |  |  |  |
| 48 | W | 1 |  |  |  |  |
| 49 | F | 1 |  |  |  |  |
| 50 | K | 1 |  |  |  |  |
| 51 | N | 1 |  |  |  |  |
| 52 | R | 1 |  |  |  |  |
| 53 | R | 1 |  |  |  |  |
| 54 | R | 1 |  |  |  |  |
| 55 | R | 1 |  |  |  |  |
| 56 | H | 0.998 | Y | 0.001 |  |  |
| 57 | K | 1 |  |  |  |  |
| 58 | I | 1 |  |  |  |  |
| 59 | Q | 0.995 | E | 0.004 |  |  |
| 60 | S | 0.996 | A | 0.001 | N | 0.001 |
|  |  | 0.986 |  |  |  |  |
