# Supplementary material for: Ancient mechanisms for the evolution of the bicoid homeodomain's function in fly development
Source: eLife. 2018 Oct 9;7:e34594. doi: 10.7554/eLife.34594 (PMC6177261; doi:10.7554/eLife.34594)

**Supplemental File 3.** **PBM binding specificity profiles are robust to the uncertainty in ancestral sequence and choice of flanking sequences.**

Energy PWMs were inferred from PBMs using maximum likelihood reconstructions (no suffix) or alternative reconstructions (suffix “altAll”) and from constructs using 15 flanking residues derived from D. melanogaster *bcd* protein (prefix “B-“) or from *D. melanogaster* Zen protein (no prefix). The Predicted column shows R^2^ for the comparison of intensities predicted from a model fit to the PBM in the column labeled “Training” against measured intensities in the PBM of the “Prediction” protein. The “Measured” column shows R^2^ for comparison of measured intensities from the training PBM against the prediction sample.


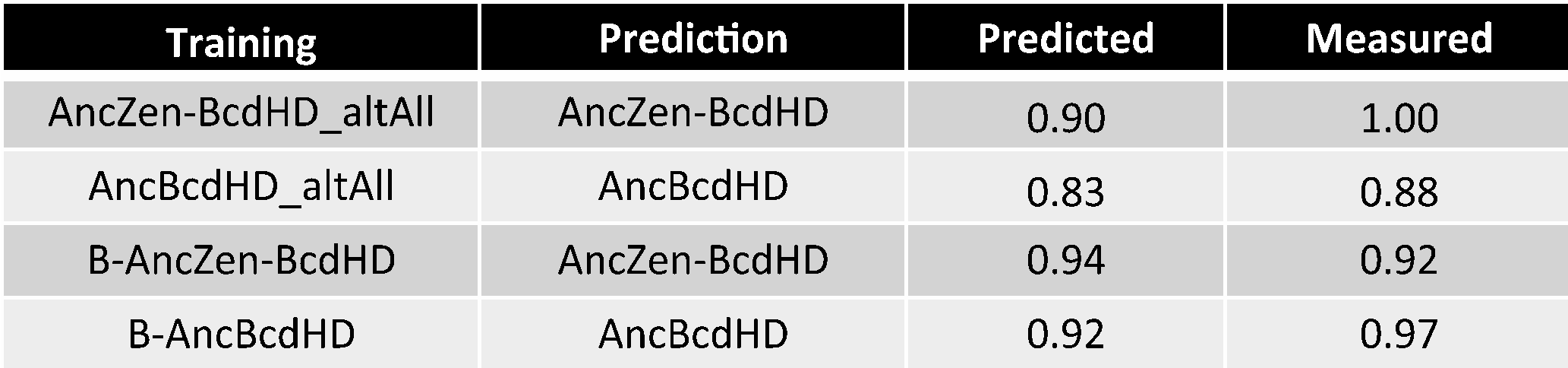

Supplement: Supplementary file 3. — Energy PWMs were inferred from PBMs using maximum likelihood reconstructions (no suffix) or alternative reconstructions (suffix ‘altAll’) and from constructs using 15 flanking residues derived from D. melanogaster Bcd protein (prefix 'B-’) or from D. melanogaster Zen protein (no prefix). The Predicted column shows R2 for the comparison of intensities predicted from a model fit to the PBM in the column labeled ‘Training’ against measured intensities in the PBM of the ‘Prediction’ protein. The ‘Measured’ column shows R2 for comparison of measured intensities from the training PBM against the prediction sample. [file elife-34594-supp3.docx]
