# Supplementary material for: Ancient mechanisms for the evolution of the bicoid homeodomain's function in fly development
Source: eLife. 2018 Oct 9;7:e34594. doi: 10.7554/eLife.34594 (PMC6177261; doi:10.7554/eLife.34594)
Supplement: Supplementary file 4. — Binding by various homeodomain constructs (HD) to canonical DNA motifs BM or ZM was measured by SPR. The estimated on-rate (kon), off-rate (koff), association constrant (KA) and average residence time t1/2 are shown. Mean and SEM of three replicates is shown for each parameter. [file elife-34594-supp4.docx]

**Supplemental Table 4**. **Kinetic binding parameters inferred by surface plasmon**

**resonance assays.** Binding by various homeodomain constructs (HD) to

canonical DNA motifs BM or ZM was measured by SPR. The estimated on-rate (k_on_), off-rate (k_off_), association constrant (K_A_) and average residence time t_1/2_ are shown.

Mean and SEM of three replicates is shown for each parameter.

| **HD** | **DNA motif** | **k_on_ (M^-1^s^-1^ x10^5^)** | **k_off_ (M^-1^s^-1^ x10^-3^)** | **K_A_ (M^-1^ x10^7^)** | **t_1/2_ (min)** |
| --- | --- | --- | --- | --- | --- |
| AncZB | BM | 10.3±1.56 | 17.1±1.36 | 5.65±0.85 | 0.68±0.25 |
| AncBcd | BM | 2.11±0.14 | 0.26±0.021 | 81.3±12.0 | 44.78±3.59 |
| AncZB | ZM | 1.05±0.13 | 1.14±0.06 | 8.70±1.33 | 10.13±0.63 |
| AncBCD | ZM | 1.19±0.22 | 3.82±0.33 | 3.05±0.31 | 3.02±0.26 |
| AncZB-q50K | BM | 1.96±0.08 | 1.51±0.16 | 12.9±1.85 | 7.65±0.79 |
| AncBcd-K50q | BM | 1.72±0.01 | 7.39±0.24 | 2.33±0.08 | 1.56±0.05 |
| AncZB-q50K | ZM | 7.38±0.2 | 31.2±1.65 | 2.38±0.06 | 0.37±0.02 |
| AncBcd-K50q | ZM | 20.4±6.9 | 6.9±0.03 | 26.2±8.91 | 1.67±0.01 |
