# Supplementary material for: Ancient mechanisms for the evolution of the bicoid homeodomain's function in fly development
Source: eLife. 2018 Oct 9;7:e34594. doi: 10.7554/eLife.34594 (PMC6177261; doi:10.7554/eLife.34594)

**Supplemental Table 5. Frequency of phenotypes in transgenic embryos.**

Frequency of observation (in percent) of various phenotypic features in cuticular preps of embryos from females of various genotypes is shown. YW, wild-type; *bcd*-, *bcd^E1^*/*bcd^E1^*; AncZBq50K, *bcd*- embryos rescued with a *bcd* construct containing the ancestral AncZB homeodomain with the q50K substitution; AncZB-K50R54, *bcd*- embryos rescued with a *bcd* construct containing the ancestral AncZB homeodomain with the q50K and m54R substitutions. No mutant larvae showed complete head development; partial head is defined as the observation of microscopic bright spots (sclerotized tissue) at or near the anterior tip. T1-T3: Thoracic segments, numbered anterior to posterior, A1-A4: Abdominal segments, numbered anterior to posterior. The number of larvae counted for each genotype, *n,* is shown.


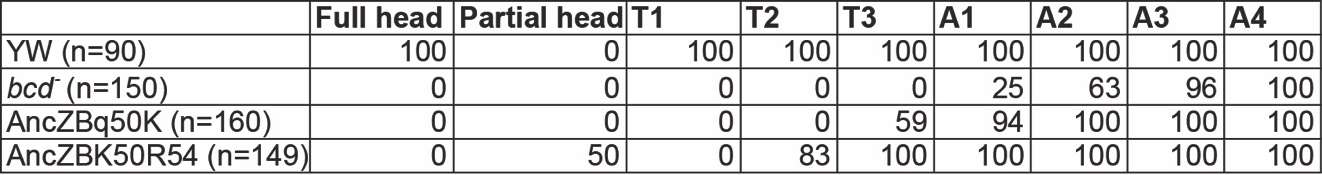

Supplement: Supplementary file 5. — Frequency of observation (in percent) of various phenotypic features in cuticular preps of embryos from females of various genotypes is shown. YW, wild-type; bcd-, bcdE1/bcdE1; AncZBq50K, bcd- embryos rescued with a bcd construct containing the ancestral AncZB homeodomain with the q50K substitution; AncZB-K50R54, bcd- embryos rescued with a bcd construct containing the ancestral AncZB homeodomain with the q50K and m54R substitutions. No mutant larvae showed complete head development; partial head is defined as the observation of microscopic bright spots (sclerotized tissue) at or near the anterior tip. T1-T3: Thoracic segments, numbered anterior to posterior, A1-A4: Abdominal segments, numbered anterior to posterior. The number of larvae counted for each genotype, n, is shown. [file elife-34594-supp5.docx]
