# Supplementary material for: Ancient mechanisms for the evolution of the bicoid homeodomain's function in fly development
Source: eLife. 2018 Oct 9;7:e34594. doi: 10.7554/eLife.34594 (PMC6177261; doi:10.7554/eLife.34594)

**Supplemental Table 6. Protein binding microarray experimental reproducibility and binding model fitting results.** The table shows model accuracy and cross-replicate reproducibility for protein binding microarrays. Each row represents one HD tested in two replicates. Energy models (see methods) were trained on data from replicate 1, used to predict the median intensity of each possible 6-mer, and compared to the experimentally measured median intensity of DNA probes carrying that 6-mer (see Methods). Column a, coefficient of determination (R^2^) for model-predicted intensities from PBM replicate 1 against measured intensities from replicate 1. Column b, R^2^ for model-predicted intensities from replicate 2 against measured intensities for replicate 2. Column c, R^2^ for measured intensities from PBM replicate 1 against measured intensities from PBM replicate 2. Cross-replicate statistical reproducibility is reduced because stripping and re-using protein microarrays reduces contrast; nevertheless, the specific binding profiles are similar between replicates (see Suppl. File 6).


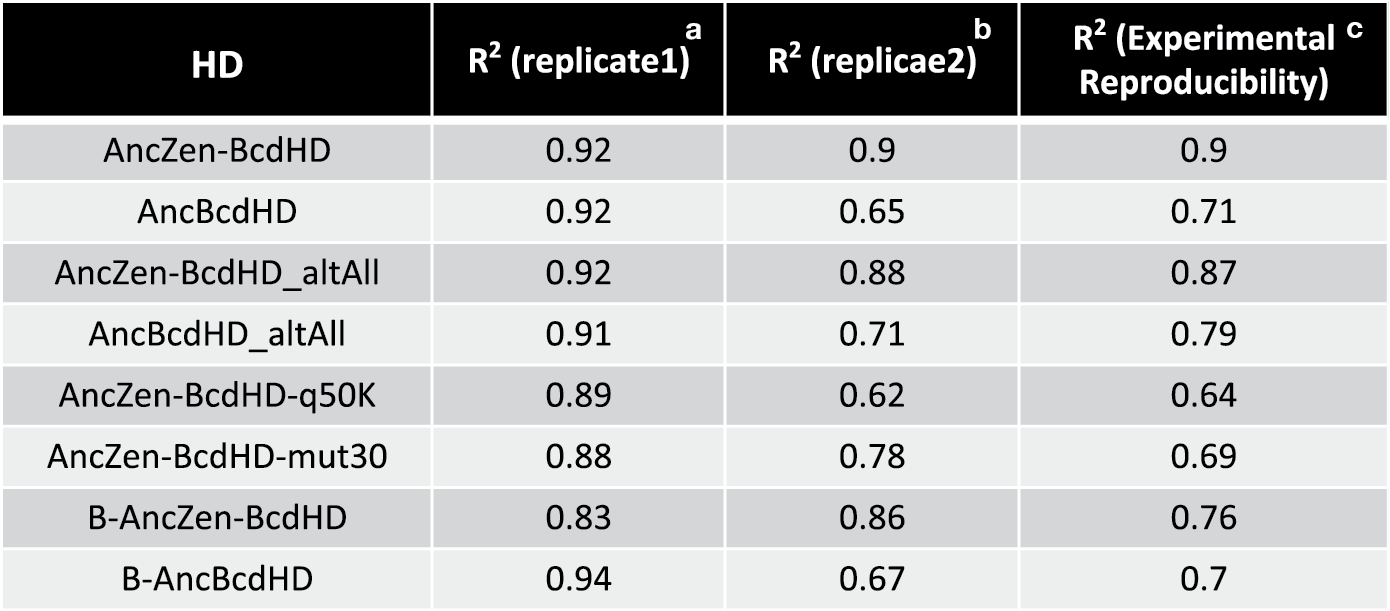

Supplement: Supplementary file 6. — The table shows model accuracy and cross-replicate reproducibility for protein binding microarrays. Each row represents one HD tested in two replicates. Energy models (see Materials and methods) were trained on data from replicate 1, used to predict the median intensity of each possible 6-mer, and compared to the experimentally measured median intensity of DNA probes carrying that 6-mer (see Materials and methods). Column a, coefficient of determination (R2) for model-predicted intensities from PBM replicate 1 against measured intensities from replicate 1. Column b, R2 for model-predicted intensities from replicate 2 against measured intensities for replicate 2. Column c, R2 for measured intensities from PBM replicate 1 against measured intensities from PBM replicate 2. Cross-replicate statistical reproducibility is reduced because stripping and re-using protein binding microarrays reduces signal; nevertheless, the specific binding profiles are similar between replicates (see Supplementary file 6). [file elife-34594-supp6.docx]
